# Spatially defined Rabi spectroscopy for uninterrupted optical clock interrogation

**DOI:** 10.1038/s41467-026-76017-1

**Published:** 2026-08-06

**Authors:** Koki Nishida, Ryoto Takeuchi, Shigenori Tsuji, Shoichi Okaba, Hidetoshi Katori

**Affiliations:** 1 Department of Applied Physics, Graduate School of Engineering, The University of Tokyo, Bunkyo-ku, Japan; 2 Spacetime Engineering Research Team, RIKEN Center for Advanced Photonics, Wako, Japan; 3 JEOL Ltd., Akishima, Japan

**Keywords:** Quantum metrology, Atom optics

## Abstract

Optical lattice clocks achieve fractional frequency uncertainties of 10⁻¹⁸, yet stability is constrained by the dead time between cooling, preparation, interrogation, and detection. This sampling aliases local-oscillator noise into the clock signal (the Dick effect) and prevents continuous accumulation of oscillator phase information. We demonstrate spatially defined Rabi spectroscopy of ultracold ⁸⁸Sr atoms continuously transported in a moving optical lattice. A longitudinal excitation geometry preserves Lamb–Dicke confinement and suppresses Doppler broadening. Clock excitation is enabled only within a localised region by a transverse magnetic mixing field, defining the atom–laser interaction in space rather than in time and decoupling interrogation from preparation and detection. Transporting atoms at 16 mm s⁻¹ through a 12-mm interaction region yields a 1.2-Hz-wide spectrum close to the transit-time Fourier limit while maintaining uninterrupted atom delivery. This approach provides a practical route toward dead-time-free optical clock interrogation of continuously delivered atomic ensembles.

## Abstract (web summary)

Recent progress in optical lattice clocks has enabled metrological advances in several fields, yet stability remains challenging. Here, the authors demonstrate spatially defined Rabi spectroscopy of ⁸⁸Sr atoms continuously transported in a moving optical lattice. The scheme yields a 1.2-Hz-wide spectrum, providing a route towards dead-time-free optical interrogation.

## Introduction

Optical lattice clocks (OLCs)^1^ have achieved fractional frequency uncertainties of 10⁻¹⁸ (refs. ^2–5^), enabling advances in relativistic geodesy^6–8^, tests of fundamental physics^9,10^, and optical timekeeping and clock networks^11,12^. An essential feature of OLCs is the use of large ensembles of neutral atoms, which efficiently suppress quantum projection noise (QPN)^13^. As QPN has been reduced, the short-term stability of optical clocks has been limited by the local oscillator’s frequency noise. As a consequence, efforts to further improve clock stability have increasingly focused on reducing thermal noise in reference cavities, including long-spacer designs^14^ and cryogenic silicon resonators^15,16^, which have achieved record-low laser frequency instability and have become a defining element of state-of-the-art optical clocks.

Despite this progress, reducing the averaging time required to achieve a given level of stability remains challenging. In current OLCs, atomic interrogation is inherently intermittent because cooling, preparation, interrogation, and detection are performed sequentially. This temporal sampling causes laser-frequency noise to be aliased into the clock signal via the dead time between interrogation cycles—the Dick effect^17^—preventing clocks from fully exploiting the atomic reference.

To achieve uninterrupted clock interrogation, where atoms monitor the local oscillator without temporal gaps, zero-dead-time (ZDT) operation has been pursued using dual-ensemble interleaving schemes^18–20^. However, continuous interrogation of an atomic stream serving as a frequency reference has not yet been experimentally demonstrated for OLCs. Uninterrupted spectroscopy has been realised for decades using Ramsey-type separated-field schemes for thermal atomic beams^21,22^, but these approaches have so far not achieved spectral resolution competitive with state-of-the-art optical clocks due to limited interaction times and residual Doppler effects.

Here, we realise spatially defined Rabi spectroscopy of continuously transported ultracold ⁸⁸Sr atoms. Atoms are delivered in a moving optical lattice, such that preparation, interrogation, and state-selective detection occur simultaneously at different positions along the transport axis. We demonstrate a longitudinal excitation geometry to suppress Doppler broadening^23,24^ for atoms in a moving lattice. Clock excitation is spatially confined by magnetically inducing the atom–laser coupling only within a localised region, enabling Fourier-limited Rabi spectroscopy under continuous interrogation. Using this approach, we demonstrate a 1.2-Hz-wide Rabi linewidth, establishing a practical route toward dead-time-free optical clock operation and related precision spectroscopy.

## Results

### Experimental concept and setup

**Fig. 1:**
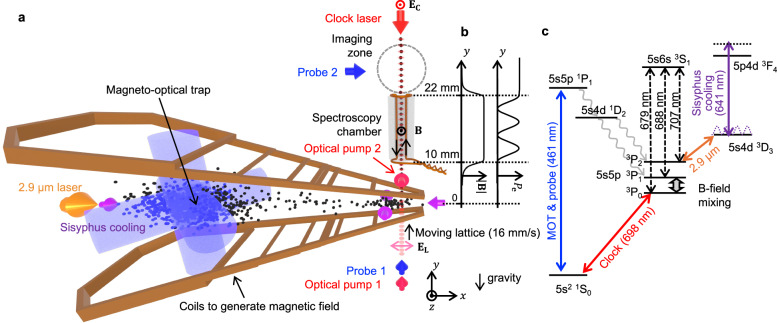
Experimental concept and setup for longitudinal clock spectroscopy under continuous atomic transport. **a** Schematic of the experimental configuration. Ultracold ⁸⁸Sr atoms are continuously cooled and guided using a magnetic guide implemented with custom multilayer printed-circuit-board (PCB) coils. Atoms are loaded into a moving optical lattice operated at the magic wavelength (813.4 nm) and transported upward at a constant velocity u. State preparation, spectroscopy, and state-selective detection are spatially separated along the transport axis. Ground-state atoms are detected via fluorescence on the ${{}^{1}S}_{0}{-}^{1}{P}_{1}$ transition using 461-nm probe light introduced either along the lattice axis (Probe 1) or transversely in the imaging region (Probe 2). The dashed circle indicates the imaging zone. ${{{\bf{E}}}}_{{{\rm{C}}}}$ and ${{{\bf{E}}}}_{{{\rm{L}}}}$ denote the electric-field vectors of the clock and lattice lasers, respectively. **b** Longitudinal spectroscopy with spatially defined excitation. A linearly polarised 698-nm clock laser counter-propagates with the moving lattice along the atomic transport axis, realising a longitudinal excitation geometry that maintains Lamb-Dicke confinement. Clock excitation is enabled only within a localised spatial region by a transverse magnetic mixing field ${{\bf{B}}}$, generated by current-carrying wires embedded inside a permalloy magnetic shield. **c** Energy-level diagram showing the ${{}^{1}S}_{0}$ ground state, the ${{}^{3}P}_{0}$ clock excited state, and magnetic-field-induced mixing with the ${{}^{3}P}_{1}$ state.

Figure 1a shows the experimental configuration developed for longitudinal clock spectroscopy under continuous atom cooling and transport. Ultracold ⁸⁸Sr atoms are continuously produced and transported using a moving optical lattice operated at the magic wavelength (${\lambda }_{{{\rm{L}}}}\approx$813.4 nm)^25^. Atoms emerging from a Zeeman slower are captured in a magneto-optical trap (MOT) operated at 461 nm, where a weak branching decay populates the metastable ${{}^{3}P}_{2}$ state^26^. Low-field-seeking atoms are magnetically guided in a tapered quadrupole field generated by custom 14-layer printed-circuit-board (PCB) coils. Narrow-line Doppler cooling on the ${{}^{3}P}_{2}{-}^{3}{D}_{3}$ transition at 2.92 µm^27^, assisted by Sisyphus cooling in the ${{}^{3}D}_{3}$ state^28^, reduces the atomic temperature to several tens of microkelvin while maintaining continuous transport.

Atoms are loaded from the magnetic guide into the lattice and transported upward at a velocity of *u* = 16 mm s^−1^, set by the frequency difference between two counter-propagating lattice beams enhanced in a triangular ring cavity. The cavity geometry provides a long Rayleigh range of 71 mm and a lattice depth of about 130 μK, enabling a continuous atomic stream to reach the spectroscopy region with an atom flux of approximately 1 × 10^5^ atoms s^−1^. To maintain Lamb-Dicke confinement for the clock laser during atomic transport, a 698-nm clock laser counter-propagates with the moving lattice along the transport axis, realising a longitudinal excitation geometry^24^.

Clock interrogation is performed as atoms traverse an interaction region with $L=12\,{{\rm{mm}}}$ enclosed by a permalloy magnetic shield, where a transverse magnetic field *B* (Fig. 1b), generated by a pair of current-carrying wires, locally admixes the forbidden ${{}^{1}S}_{0}{-}^{3}{P}_{0}$ clock transition with the dipole-allowed ${{}^{3}P}_{1}$ state. For clock laser intensity ${I}_{{\rm{c}}}$, polarised parallel to the magnetic field, the on-resonance Rabi frequency is given by $\Omega \left(B\right)=\alpha B\sqrt{{I}_{{{\rm{c}}}}}$ with α the magnetically induced coupling coefficient^29^. As a result, atoms experience coherent Rabi coupling solely while traversing this field-defined spatial window. Owing to the longitudinal, Lamb-Dicke-confined excitation, Doppler broadening of the spectrum is strongly suppressed.

State-selective detection is carried out in an imaging zone (dashed circle in Fig. 1a) downstream of the spectroscopy chamber. Ground-state atoms are detected via fluorescence on the ${{}^{1}S}_{0}{-}^{1}{P}_{1}$ transition using a 461-nm probe beam introduced either along the moving lattice (Probe 1) or transversely in the imaging region (Probe 2). Atoms in the excited ${{}^{3}P}_{0}$ state are optically pumped to the ground state via the ${{}^{3}S}_{1}$ level using 679- and 707-nm light and subsequently detected in the same manner. Relevant energy levels are shown in Fig. 1c. Fluorescence images recorded with an electron-multiplying CCD (EMCCD) camera provide spatially resolved populations of ground- and excited-state atoms, from which we directly observe spatially encoded Rabi oscillations that reveal the quantum evolution of atoms under continuous transport. During imaging, the 461-nm light used for the cooling is switched off to improve the signal-to-noise ratio.

### Spatially encoded Rabi oscillations

**Fig. 2:**
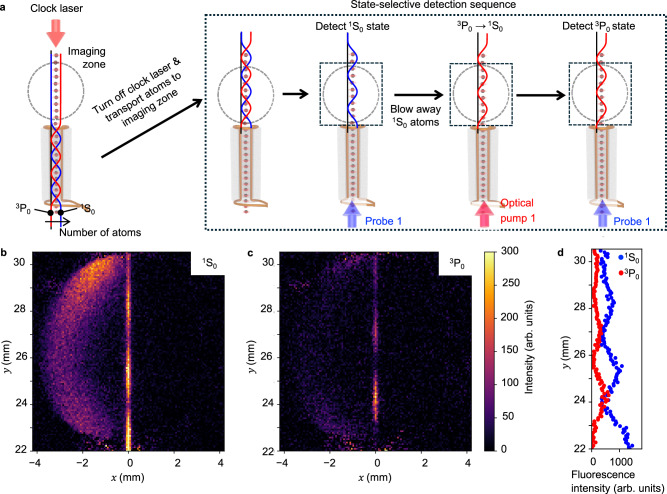
State-selective imaging of spatially encoded Rabi oscillations. **a** Timing sequence for clock excitation and fluorescence detection. Atoms undergo Rabi excitation only while traversing the spatially defined interaction region and are transported to an imaging zone (dashed circle). By turning off the clock laser at a chosen time and allowing the atoms to propagate 12 mm downstream, we record a snapshot of the state evolution within the spectroscopy chamber. State-selective fluorescence imaging is conducted by applying a 461-nm probe laser (Probe 1, shown by blue arrows) to measure ground-state atoms ${N}_{\rm{g}}$. After heating the atoms away from the lattice, excited-state atoms ${N}_{\rm{e}}$ are optically pumped to the ground state by applying 679- and 707-nm lasers (Optical pump 1, shown by a red arrow), and the ground state population is measured by Probe 1. **b** Fluorescence image of ground-state atoms. A semicircular image on the left is due to the atomic fluorescence reflected by a concave mirror behind the atoms. **c** Fluorescence image of excited-state atoms. **d** One-dimensional population profiles along the transport axis. The ${{}^{1}S}_{0}$ (blue) and ${{}^{3}P}_{0}$ (red) populations exhibit clear out-of-phase oscillations imprinted in space, providing a direct record of the Rabi evolution experienced as atoms passed through the spectroscopy chamber.

Building on the experimental geometry described above, we first visualise Rabi dynamics encoded in space as atoms traverse the localised excitation region. Figure 2a shows the measurement sequence used to map the internal-state evolution onto the atomic position.

Ultracold ⁸⁸Sr atoms are continuously transported by the moving optical lattice through the spectroscopy region, where clock excitation is magnetically enabled only within a finite spatial window. To take a snapshot of the internal-state evolution, we turn off the clock laser at a chosen time and let the atoms propagate 12 mm downstream to the imaging zone, where the spatially encoded populations are read out. With a constant transport velocity u, the atomic position $y={ut}+{y}_{0}$ maps directly onto the interaction time t, such that the spatial modulation encodes conventional time-domain Rabi dynamics (Fig. 2a).

Figures 2b, c show representative fluorescence images of atoms detected in the ground and excited clock states, respectively. Clear, out-of-phase oscillations are imprinted along the transport direction, indicating coherent population transfer as atoms traverse the interaction region. By integrating the fluorescence signal transverse to the transport axis, we obtain one-dimensional population profiles (Fig. 2d), providing a direct spatial record of the Rabi evolution. State-selective fluorescence images are converted to atom numbers ${N}_{\rm{g}}$ and ${N}_{\rm{e}}$ using the calibrated fluorescence collection efficiency and the state-pumping efficiency ${{}^{3}P}_{0}\to {{}^{1}S}_{0}$, which are used to obtain line density of atoms ${n}_{1{{\rm{D}}}}(y)$ and excitation probability ${P}_{{{\rm{e}}}}\left(y\right)={N}_{\rm{e}}/({N}_{\rm{g}}+{N}_{\rm{e}})$.

The gradual reduction in oscillation amplitude along the transport direction arises from background gas collisions, lattice-induced parametric heating, and Raman transitions^30^, which are independently characterised and accounted for in the analysis (Methods). These data validate the position-to-time mapping and enable quantitative extraction of the magnetically induced coupling used for the Fourier-limited spectroscopy presented below.

### Quantitative analysis of Rabi dynamics and magnetically induced coupling

**Fig. 3:**
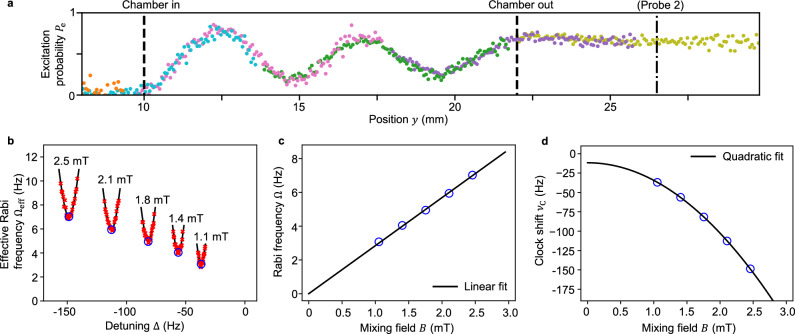
Determination of Rabi dynamics and clock shifts from spatially encoded excitation profiles. **a** Excited-state probability ${P}_{{{\rm{e}}}}(y)$ along the transport axis, obtained by combining multiple images indicated by different colours, which are unrelated to the colour scheme used in (**b–d**). The clock excitation was performed at clock-laser intensity *I*_c_ = 0.76 W cm^−2^ and the mixing field B=0.7 mT. We analysed spatial oscillation of ${P}_{{{\rm{e}}}}(y)$ to obtain the effective Rabi frequency ${\Omega }_{{{\rm{eff}}}}\left(\Delta \right)$. **b** ${\Omega }_{{{\rm{eff}}}}\left(\Delta \right)$ versus clock-laser detuning Δ for different mixing fields B measured at clock-laser intensity *I*_c_ = 0.56 W cm^−2^ (red crosses). Fitting ${\Omega }_{{{\rm{eff}}}}\left(\Delta \right)$ provides the on-resonance Rabi frequency Ω(B) and the clock shift ${\nu }_{{{\rm{c}}}}$ (blue circles). **c** On-resonance Rabi frequency Ω(B) (blue circles) as a function of the mixing field B. The solid line shows a linear fit. Linear scaling confirms magnetic-field-induced admixture of the clock state. **d** Extracted clock shift ${\nu }_{{{\rm{c}}}}$ (blue circles) versus B. The solid line shows a quadratic fit. The quadratic dependence reflects the second-order Zeeman shift. Extrapolating B→0 yields the residual clock shift of ${\nu }_{{{\rm{c}}}}=-11.7\left(2\right){\mbox{Hz}}$, consistent with the sum of clock-light shift, the BBR shift, and 461-nm light shift.

By combining successive images taken under identical interrogation conditions, we reconstruct the excitation probability ${P}_{{{\rm{e}}}}(y)$ over an extended spatial range as shown in Fig. 3a, including both upstream and downstream regions relative to the spectroscopy chamber. At y=7 mm, atoms were optically pumped to the ground state by applying lasers at 679 and 707 nm (Optical pump 2 in Fig. 1a). These atoms then entered the spectroscopy chamber at y=10 mm, where they underwent Rabi oscillations until exiting the chamber at *y* = 22 mm. As the state-selective fluorescence detection is performed downstream of the interaction region, the measured fluorescence intensity profiles include the loss of atoms during transport. In particular, the larger loss rate of the ${{}^{3}P}_{0}$ state during transport would otherwise underestimate the excitation probability. We therefore correct these spatial profiles using independently measured state-dependent loss rates (see Methods).

 The observed spatial oscillations are described by an effective Rabi frequency ${\Omega }_{{{\rm{eff}}}}\left(\Delta \right)=\sqrt{{\Omega {\left(B\right)}^{2}+\left(\Delta -{\nu }_{{\rm{c}}}\right)}^{2}}$, where Ω(B) is the magnetically induced on-resonance Rabi frequency, Δ is the clock-laser detuning with respect to the unperturbed ^88^Sr clock transition^25^ excluding the moving-lattice Doppler shift ${\nu }_{{{\rm{D}}}}\approx 23.3\,{{\rm{kHz}}}$. ${\nu }_{{{\rm{c}}}}$ denotes the total clock shift, which includes Zeeman shift ${\delta }_{B}$, clock-light shift ${\delta }_{{{\rm{c}}}}$, blackbody radiation (BBR) shift ${\delta }_{{{\rm{BBR}}}}$, and 461-nm cooling-laser-induced light shift ${\delta }_{461}$. Figure 3b shows the detuning dependence of the spatially encoded effective Rabi frequency ${\Omega }_{{{\rm{eff}}}}$ for several magnetic-field strengths, which allow extraction of both Ω(B) and ${\nu }_{{{\rm{c}}}}$.

Figure 3c shows the extracted $\Omega (B)(=\alpha B\sqrt{{I}_{{{\rm{c}}}}})$ as a function of the applied magnetic field B for clock-laser intensity ${I}_{{{\rm{c}}}}=0.56$ ${{\rm{W}}}$ ${{\rm{c}}}{{{\rm{m}}}}^{-2}$. The resulting linear dependence indicates that the coupling strength is given by magnetic-field-induced admixture of the clock state, as expected for magnetically enabled clock excitation. Figure 3d shows the total clock shift ${\nu }_{{{\rm{c}}}}$ as a function of magnetic-field strength, exhibiting the second-order Zeeman shift ${\delta }_{B}=\beta {B}^{2}$. Extrapolating B→0 removes the Zeeman shift and yields the residual shift given by the sum of ${\delta }_{{{\rm{c}}}}$, ${\delta }_{{{\rm{BBR}}}}$, and ${\delta }_{461}$.

### Clock spectroscopy with Fourier-limited resolution

**Fig. 4:**
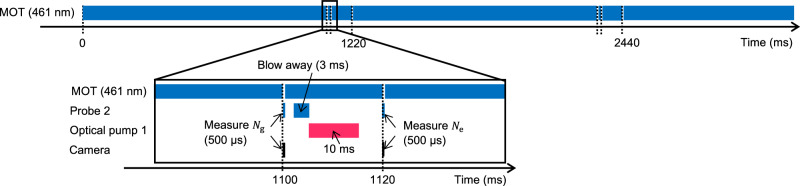
Schematic timing sequence for the clock-spectrum measurement. The 813-nm moving lattice lasers, 698-nm clock laser, Optical pump 2 lasers (679 and 707 nm), 641-nm Sisyphus-cooling light, and 2.9-µm infra-red-cooling light, 461-nm Zeeman-slower light are kept on throughout the sequence. The MOT light remains on during clock interrogation so that its clock shift and excitation are included in the measured spectrum, while it is switched off only during the brief Probe−2 fluorescence sequence to suppress stray cooling light on the EMCCD camera. The lower panel shows the essential state-selective detection sequence at Probe 2. Three 500-µs-long Probe−2 pulses are applied for determining the excitation probability ${P}_{\rm{e}}$: the first measures the ground-state atom number ${N}_{\rm{g}}$, the second measures the excited-state atom number ${N}_{\rm{e}}$ after blowing atoms away by Probe 2 and repumping by Optical pump 1, and the third records the EMCCD background after removal of the remaining atoms (at 1220 ms, which is not shown in the lower panel). Camera triggers are shown by black ticks. The interval between the ${N}_{\rm{g}}$ and ${N}_{\rm{e}}$ measurements is 20 ms. The 1220-ms cycle time is set mainly by the transport time from the upstream optical-pumping position to Probe 2 at *u* = 16 mm s^−1^. The clock-laser detuning is held fixed during each acquisition and stepped between measurements. Each data point in Fig. 5 represents the average of five acquisitions.

Finally, we demonstrate clock spectroscopy for the continuously transported atomic stream. During clock excitation, the moving lattice, cooling light, Optical pump 2, and clock laser remained on. For each data point, the clock-laser detuning Δ was set to a fixed value and held constant during acquisition, and the detuning was stepped by 0.1 Hz between measurements. The excitation probability ${P}_{{{\rm{e}}}}$ was measured with Probe 2 (beam diameter $\phi \approx 1$ mm) at y=26.5 mm, which is d=4.5 ${{\rm{mm}}}$ downstream of the interrogation region (see Fig. 1a). To suppress stray 461-nm light on the EMCCD camera, only the 461-nm MOT light was switched off during the brief Probe-2 fluorescence sequence; this technical interruption occurred after clock interrogation and therefore did not remove the 461-nm light shift and decoherence accumulated during excitation. The timing sequence with a repetition period of 1220 ms and the state-selective detection of ${N}_{\rm{g}}$ and ${N}_{\rm{e}}$ is shown in Fig. 4.

**Fig. 5:**
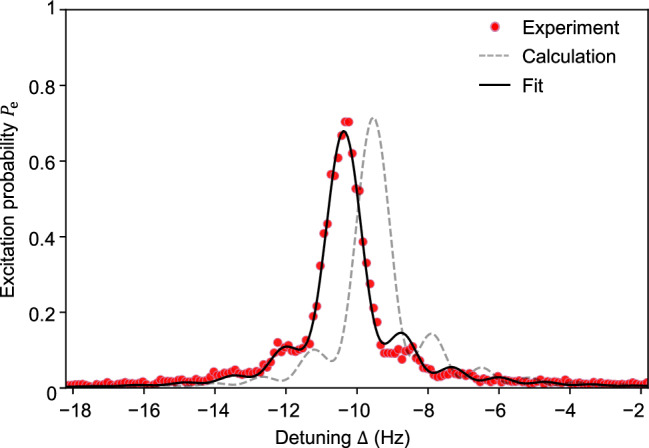
Clock spectroscopy with Fourier-limited resolution. Excitation probability on the ${{}^{1}S}_{0}{-}^{3}{P}_{0}$ clock transition measured as a function of clock-laser detuning. Atoms are continuously transported through the spatially defined excitation region. The clock laser and cooling beams remain on during interrogation, and the excitation probability is recorded in the downstream imaging zone by Probe 2; the 461-nm MOT light is switched off only during the brief Probe−2 fluorescence sequence. The spectrum exhibits a 1.2-Hz FWHM, consistent with the Fourier limit set by the finite interaction time determined by the excitation length and transport velocity. The dashed line is a model using independently determined parameters (Methods). The solid line shows the fit to the data with the centre frequency −10.4 Hz and a dephasing rate of ${\gamma }_{\perp }=2.3\,{{{\rm{s}}}}^{-1}$.

Figure 5 shows the excitation spectrum exhibiting a full width at half maximum (FWHM) of 1.2 Hz. This width is mainly set by the transit-time-limited Fourier width for a rectangular interaction window, $0.886/\tau \approx 1.18\,{{\rm{Hz}}}$, where the atomic transit time is τ(=L/u)=0.75 s for an interaction length $L=12\,{{\rm{mm}}}$ and a transport velocity *u* = 16mm s^−1^. These results demonstrate that the longitudinal geometry and spatially defined excitation provide a robust and quantitatively understood platform for continuous, Fourier-limited interrogation of moving atoms. No additional post-processing correction for population loss between the spectroscopy chamber and Probe 2 was applied to the experimental data in Fig. 5. Outside the chamber, the measured loss rates are nearly equal, ${\gamma }_{\rm{S}}^{\left({{\rm{out}}}\right)}=1.7(1)\,{{{\rm{s}}}}^{-1}$ and ${\gamma }_{\rm{P}}^{\left({{\rm{out}}}\right)}=1.93(9)\,{{{\rm{s}}}}^{-1}$ for the clock states, so the differential relaxation mainly affects the overall signal amplitude and has negligible effect on the spectral peak position within the present uncertainty.

The dashed line in Fig. 5 represents a calculation based on independently measured parameters, including the in- and out-of-chamber state-dependent loss rates, the spatial magnetic-field profile, the extracted Rabi frequency (Fig. 3), excitation by stray 461-nm photons, and the clock shift, as summarised in Table 1 and in Methods. The solid line shows the fit to the observed spectrum to estimate a centre frequency ${\nu }_{{{\rm{c}}}}=-10.4$ Hz and a dephasing rate ${\gamma }_{\perp }=2.3\,{{{\rm{s}}}}^{-1}$ to account for side-lobe suppression.

**Table 1:** Uncertainty budget for the clock spectrum

Effect	Correction (Hz)	Uncertainty (Hz)
Lattice Doppler shift, ${\nu }_{{{\rm{D}}}}$	(-23292.50)	0.01
2nd order Zeeman shift, ${\delta }_{B}$	−4.57	0.03
Clock light shift, ${\delta }_{{{\rm{c}}}}$	-3.1	0.4
Lattice light shift	0	0.1
BBR shift, ${\delta }_{{{\rm{BBR}}}}$	−2.33	0.06
461-nm-light shift, ${\delta }_{461}$	+0.40	0.1
Total (excluding Doppler shift), ${\nu }_{{{\rm{c}}}}$	-9.6	0.5

The lattice Doppler correction is determined by the frequency difference of the lattice lasers $\Delta {\nu }_{{{\rm{L}}}}=40\,{{\rm{kHz}}}$, and is subtracted in advance. The lattice laser, operating at an intensity of 160 kW cm^-2^, is tuned to the magic frequency within 10 MHz. Clock excitation was performed with a laser intensity *I*_c_ = 0.18 W cm^−2^ and a mixing field $B=0.45\,{\mbox{mT}}$. A possible density-dependent shift/broadening (discussed in the main text) is not included in this budget.

The additional 0.8-Hz redshift and the excess broadening relative to the dashed line may be partly due to a DC Stark shift from stray static electric fields in the spectroscopy chamber, where the Kapton-coated wires running close to the atoms may generate patch potentials. An electric field of 4.6 V cm^−1^ would be sufficient to explain the observed shift and could also contribute to inhomogeneous broadening.

As a secondary contribution, collisional effects, which are known to be pronounced in bosonic ⁸⁸Sr clocks^25,31^ may also cause redshift and broadening. From the peak atomic flux ($1\times {10}^{5}\,{{\rm{atoms}}}\,{{{\rm{s}}}}^{-1}$; see Methods), we estimate a mean occupancy of about 2.5 atoms per lattice site. A detailed characterisation of the density shift and the associated broadening will be presented elsewhere. To mitigate the collisional effects, a Laguerre-Gaussian lattice with multiple trapping sites^32^ offers a possible route to reducing site occupancy.

The residual 461-nm Zeeman-slower/MOT stray field present during clock excitation contributes to the clock light shift of about 0.4 Hz, as quantified by the spectra measured with and without the 461-nm light during interrogation, which implies an excitation rate ${\gamma }_{461}=0.98\,{{{\rm{s}}}}^{-1}$ for the ${{}^{1}S}_{0}$ state, causing an additional dephasing of ${\gamma }_{461}/2$ (Methods). Further reduction of stray light can be achieved by blackening the vacuum-chamber walls^33^ and employing low-scattering optical components.

For example, uninterrupted state-selective readout could be implemented by overlapping the repump beams (679/707 nm) with Probe 2 and square-wave modulating the repump light at a frequency $f\gg u/\phi (\approx 16\,{{\rm{Hz}}})$. The fluorescence signals can be time-multiplexed to separately obtain the ground- and excited-state populations. This would allow time-resolved monitoring of the excitation probability ${P}_{{{\rm{e}}}}(t)$ at an update rate set by f (for example, ~1 kHz). The achievable feedback bandwidth, however, would be limited not by f itself but by the finite atomic response time τ=L/u, which suppresses response to frequency fluctuations above order 1/τ, and by the downstream readout delay ${\tau }_{d}=d/u$, which adds phase lag to the servo. This latency ${\tau }_{d}$ is not intrinsic and can be reduced by increasing u or reducing d. Developing a robust clock-error signal and feedback architecture that avoids offset due to line-shape drifts is beyond the scope of the present work.

In a fully uninterrupted fluorescence-readout implementation, resonant photons generated during Probe-2 state detection could constitute an additional technical perturbation if they propagate back to the spectroscopy region. This effect was not separately quantified in the present work, but it can impose a practical lower bound on the detection distance d in a fluorescence-based scheme. Such back-propagating probe light can be suppressed by choosing the Probe-2 polarisation so that the dipole radiation pattern has a node along the atomic-transport direction. For example, a linearly y-polarised probe reduces fluorescence emitted along the y-axis. Ultimately, resonant fluorescence detection could be replaced by cavity-enhanced dispersive readout^34^, in which the atomic population is inferred from the atom-induced phase or resonance-frequency shift of a cavity mode, with greatly reduced free-space photon scattering.

## Discussion

Our results demonstrate spatially defined Rabi spectroscopy, with clock excitation confined to a magnetic-field-defined spatial window along a continuously transported lattice stream. Preparation, interrogation, and detection proceed simultaneously at separated positions, and the interaction length sets the effective interrogation time and thus the Fourier-limited spectral linewidth. This geometry enables ZDT spectroscopy without relying on the temporal sequencing of multiple clocks^18–20^.

A central implication of this architecture is its impact on clock stability and oscillator requirements. In pulsed OLCs, dead time leads to Dick-effect aliasing and an instability that typically averages down as ${t}_{{{\rm{av}}}}^{-1/2}$ (ref. ^17^) for an averaging time ${t}_{{{\rm{av}}}}$. In contrast, a continuously delivered system can approach a unity interaction duty cycle, strongly suppressing Dick aliasing. The usable feedback bandwidth is nevertheless constrained by the finite atomic response time τ=L/u and the transport delay d/u between interrogation and detection. If the dominant local-oscillator noise lies within this bandwidth, the instability could in principle approach ${t}_{{{\rm{av}}}}^{-1}$-like behaviour over an intermediate range of averaging times, before crossing over to the usual ${t}_{{{\rm{av}}}}^{-1/2}$ behaviour set by quantum projection noise or technical noise. In particular, a two-zone architecture combining short and long interrogation zones^24^ provides both a wide frequency capture range and a low-QPN instability limit, potentially relaxing local-oscillator pre-stabilisation requirements and supporting more compact optical-clock architectures.

Beyond optical clocks, continuous preparation of cold atoms together with simultaneous, uninterrupted measurement constitutes a key enabling element for quantum platforms, including superradiant lasers^35,36^, scalable neutral-atom quantum processors^37^, and atom-interferometric inertial sensors^38,39^. In these systems, dead time directly limits bandwidth and aliases technical noise, while spatial separation of preparation, coherent manipulation, and readout provides a practical route to continuous operation without stringent timing synchronisation.

At a more general level, confining coherent coupling with static spatial fields provides a strategy for programming interactions geometrically along a particle’s trajectory^40–42^. This approach is naturally compatible with guided or transported platforms, such as moving optical lattices^43^ and chip-based atomic architectures^44,45^. Tailoring spatial profiles of magnetic fields enables prepare-interrogate-detect functionality within a continuous flow and allows robust quantum control through engineered in-path coupling profiles.

In conclusion, we have demonstrated transit-time-limited Rabi spectroscopy of ultracold strontium atoms continuously transported in a moving optical lattice. Longitudinal excitation in the Lamb–Dicke regime, together with a magnetically enabled interaction window, yields 1.2-Hz-wide, Fourier-limited spectra under continuous operation of an atomic stream. By spatially separating preparation, interrogation, and detection, this platform enables uninterrupted clock interrogation without sacrificing resolution and provides a basis for implementing dead-time-free clock stabilisation in future work.

## Methods

### Continuous generation of laser-cooled atoms

A thermal beam of ⁸⁸Sr is produced from an oven heated to 660 K and decelerated in a Zeeman slower operating on the 461 nm transition ($\Gamma /2\pi=32$ MHz). Atoms are captured in a magneto-optical trap at 461 nm (blue MOT), where a small branching decay populates the metastable ${{}^{3}P}_{2}$ state with a magnetic moment of $3{\mu }_{\mathrm{B}}$ (${\mu }_{\mathrm{B}}$ is the Bohr magneton). Low-field-seeking atoms in this state are magnetically trapped in an elongated spherical-quadrupole field generated by 14-layer PCB coils, which function both as MOT coils with typical magnetic field gradients of 0.9, 2.5, 3.4 mT cm^‒1^ in the *x*, *y*, and *z* directions, respectively, and as a tapered magnetic guide (see Fig. 1a, right side) along the *x*-axis to transport atoms in the lattice loading region.

In the magnetic guide, the atoms are Doppler cooled on the ${{}^{3}P}_{2}{-}^{3}{D}_{3}$ transition at 2.92 μm with a linewidth of 57 kHz. The cooling laser, blue-detuned by 5.6 MHz from the atomic resonance, propagates along the *x* direction and pushes atoms toward the right end of the magnetic guide, where they are magneto-optically cooled and trapped^27,46^. Narrow-line-mediated Sisyphus cooling is implemented on the ${{}^{3}D}_{3}{-}^{3}{F}_{4}$ transition at 641 nm (ref. ^28^) using two orthogonal standing-waves: one blue-detuned by 7 GHz creates a 30 μK lattice potential along the *x*-axis, while the other, blue-detuned by 400 MHz, creates a transverse periodic potential of 130 μK. The combined cooling process, both Doppler cooling and narrow-line Sisyphus cooling, reduces the atomic temperature to about 20 μK.

### Atom transport by the moving lattice formed inside a ring cavity

The moving optical lattice is generated inside a power-build-up triangular ring cavity with a round-trip length of 200 mm, composed of two flat mirrors and one concave mirror with a curvature radius of 150 mm. The cavity provides a power-enhancement factor of approximately 85 and a linewidth of about 7.5 MHz, enabling resonant coupling of moving lattice beams. The cavity length is actively stabilised to one of the lattice lasers using a piezoelectric transducer mounted on a mirror.

The cavity mode provides a Rayleigh range of 71 mm with its beam waist located at 55 mm away from the loading position. The ${e}^{-2}$ beam radius is 171 μm at the loading position (y=0) and gradually converges along the direction of atomic transportation, reaching $161\,{{\upmu}}{{\rm{m}}}$ ($y=10\,{{\rm{mm}}}$) and $150\,{{\upmu}}{{\rm{m}}}$ ($y=22\,{{\rm{mm}}}$) at the entrance and exit of the spectroscopy chamber, respectively. The lattice depth is about 130 μK (≈780 ${E}_{{{\rm{R}}}}$, where ${E}_{{{\rm{R}}}}$ is the lattice photon recoil energy) in the middle of the chamber at $y=16\,{{\rm{mm}}}$. Typical atom flux is about $1\times {10}^{5}\,\rm{atoms}$ ${{{\rm{s}}}}^{-1}$ at the entrance of the chamber, which was estimated from the fluorescence-calibrated one-dimensional density ${n}_{1{{\rm{D}}}}(y)$ multiplied by the transport velocity *u* (ref. ^43^). The absolute fluorescence-to-atom-number conversion was obtained from the known 461-nm scattering rate and the calibrated collection efficiency of the imaging system. Typical lattice trap decay rate for the ${{}^{1}S}_{0}$ atoms, including background-gas-collision loss and parametric heating loss, was measured to be 0.9 s^−1^ at the background gas pressure 6 × 10^−7^ Pa.

The moving optical lattice captures ultracold ${{}^{3}P}_{2}$ atoms from the magnetic guide and transports them upward at a velocity of $u={\lambda }_{{{\rm{L}}}}\Delta {\nu }_{{{\rm{L}}}}/2=16$ mm s^−1^, which is given by the frequency difference $\Delta {\nu }_{{{\rm{L}}}}=40\,{{\rm{kHz}}}$ between the moving lattice lasers with ${\lambda }_{{{\rm{L}}}}=813.4\,{{\rm{nm}}}$. This atomic motion causes the Doppler shift of ${\nu }_{\rm{D}}=({\lambda }_{{{\rm{L}}}}/{\lambda }_{{{\rm{c}}}})(\Delta {\nu }_{{{\rm{L}}}}/2)\approx 23.3\,{{\rm{kHz}}}$, for the clock laser at ${\lambda }_{{{\rm{c}}}}=698.4\,{{\rm{nm}}}$.

 Note that the Doppler shift can be accurately corrected, as the lattice frequency difference $\Delta {\nu }_{{{\rm{L}}}}$ determines the atom velocity and relevant Doppler shift. The alignment between the clock laser and the lattice axis governs the residual Doppler effect. For small angular deviations $(\theta \ll 1)$, the Doppler shift scales as ${\nu }_{{{\rm{D}}}}\cos \theta \approx {\nu }_{{{\rm{D}}}}(1-{\theta }^{2}/2)$. The alignment can be optimised to sub-milliradian precision $(\theta \sim {10}^{-4})$ by mapping and minimising the resulting parabolic dependence of the excitation frequency on the incident angle, thereby reducing the associated uncertainty to the sub-millihertz level. For the uncertainty budget (Table 1), we take $\theta=1\,{{\rm{mrad}}}$.

### Clock laser system and phase noise cancellation for the moving lattice

A waveguide periodically poled lithium niobate (WG-PPLN) crystal generates 9 mW of 698-nm clock light by injecting 40 mW of 1397-nm light, which is stabilised to a 40-cm-long reference cavity made of ultra-low expansion (ULE) glass. One-second stability of the clock laser is about $2\times {10}^{-16}$ and is stabilised to the ^87^Sr optical lattice clock with a servo time constant of several seconds^9^. The clock laser is delivered via a Doppler-noise-cancelled optical fibre. To suppress Doppler noise arising from relative phase fluctuations between the clock laser and the lattice lasers, a wedged partial reflector is placed $\approx \,$ 8 mm behind the input coupler mirror of the triangular lattice cavity. The partial reflector retro-reflects the clock laser and one of the lattice lasers, providing a common phase reference surface for the clock laser and the moving lattice.

### Optical pumping and the mixing magnetic field

Atoms are optically pumped into the ${{}^{1}S}_{0}$ ground state before entering the spectroscopy chamber. Population in the ${{}^{3}P}_{2}$ and ${{}^{3}P}_{0}$ states is pumped to the ${{}^{3}S}_{1}$ state by applying 707 and 679 nm pumping lasers, from which atoms decay to the ground state.

Magnetically induced clock excitation is realised by applying a static transverse magnetic field B. The field is generated by two copper wires separated by $\approx \,$ 2 mm and embedded inside a permalloy cylinder with an outer diameter of 5 mm. Anti-parallel currents through the wires produce an approximately uniform magnetic field of about 1 mT over the 12-mm-long interaction region. Finite-element method (FEM) simulations yield a magnetic-field conversion factor of *B*/*I* = 1.40 ${{\rm{mT}}}$ A^−1^. Where *I* is the current in each wire. Taking the second-order Zeeman shift coefficient *β* = −23.3 MHz T^−2^ (ref. ^29^), we experimentally determine *B*_exp_/*I* = 1.39(1) mT A^−1^ in good agreement with the FEM result.

### State-dependent atomic loss during transport

**Fig. 6:**
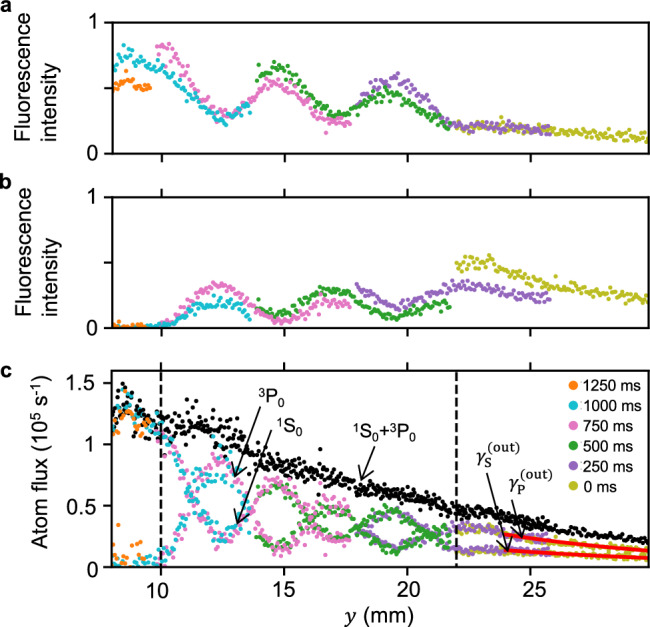
Spatial profile of atomic populations and atom flux. **a** Fluorescence intensity profiles of ${{}^{1}S}_{0}$ atoms recorded for different transport times from τ=0 to $1250\,{{\rm{m}}}{\mbox{s}}$ in steps of 250 ${{\rm{ms}}}$, indicated by different colours. **b** Same as (**a**), but for ${{}^{3}P}_{0}$ atoms. **c** Estimated atom flux in the ${{}^{1}S}_{0}$ $({{}^{3}P}_{0})$ states during transport, as a function of location *y*. Total atom flux is shown by black dots. Local overlap fitting between successive spatial profiles in **a** and **b** yielded state-dependent loss rates, which are used to determine spatial profiles in the range of $8\,{{\rm{mm}}}$ $< y < 30\,{{\rm{mm}}}$ and the excitation probability ${P}_{{{\rm{e}}}}(y)$ in Fig. 3a. Outside the spectroscopy chamber $(y > 22\,{{\rm{mm}}})$, we observed an increased loss rate of ${\gamma }_{{{\rm{S}}}}^{({{\rm{out}}})}=1.7(1)\,{{{\rm{s}}}}^{-1}$, while the ${{}^{3}P}_{0}$ loss rate ${\gamma }_{{{\rm{P}}}}^{({{\rm{out}}})}=1.93(9)\,{{{\rm{s}}}}^{-1}$ remains unchanged. These loss rates are obtained from data fitting, as shown by the red lines.

To reconstruct a consistent excitation profile over a 22-mm distance (Figs. 3a, 6), we correct the measured ${{}^{1}S}_{0}$ and ${{}^{3}P}_{0}$ profiles for state-dependent loss during transport, assuming exponential decay of each state. Inside the spectroscopy chamber, the best overlap between successive images is obtained with loss rates of ${\gamma }_{{{\rm{S}}}}=1.03\left(3\right)\,{{\mbox{s}}}^{-1}$ for the ${{}^{1}S}_{0}$ state and ${\gamma }_{{{\rm{P}}}}=1.93\left(4\right)\,{{\mbox{s}}}^{-1}$ for the ${{}^{3}P}_{0}$ state. The larger loss rate in ${{}^{3}P}_{0}$ is caused by lattice-induced Raman scattering out of ${{}^{3}P}_{0}$ for lattice depth of 780 *E*_R_ (ref. ^30^), which includes ${\gamma }_{{{\rm{R}}}1}=0.4\,{{\mbox{s}}}^{-1}$ and ${\gamma }_{{{\rm{R}}}2}=0.2\,{{\mbox{s}}}^{-1}$ to the ${{}^{3}P}_{1}$ and ${{}^{3}P}_{2}$ states, respectively, and enhanced collisional loss in ${{}^{3}P}_{0}$ (refs. ^30,47,48^). The ${}^{3}P_{0}$ profiles are reconstructed assuming a single-exponential decay $\exp (-{\gamma }_{{{\rm{P}}}}\tau )$, while the ${}^{1}S_{0}$ profiles are corrected for the influx due to lattice-induced Raman population transfer given by ${\gamma }_{{{\rm{R}}}1}$.

Outside the chamber, we observe an increased ground-state loss rate of ${\gamma }_{{\rm{S}}}^{({{\rm{out}}})}=1.7(1)\,{{\mbox{s}}}^{-1}$, while the excited-state loss rate remains unchanged within uncertainty, ${\gamma }_{{\rm{P}}}^{({{\rm{out}}})}=1.93\left(9\right)\,{{\mbox{s}}}^{-1}\approx {\gamma }_{{{\rm{P}}}}.$ We attribute the excess ground-state loss rate $\Delta {\gamma }_{{{\rm{S}}}}={\gamma }_{{\rm{S}}}^{({{\rm{out}}})}-{\gamma }_{{{\rm{S}}}}=0.67(10)\,{{\mbox{s}}}^{-1}$ to heating atoms out of the lattice caused by stray 461‑nm Zeeman‑slower and MOT light on the ${{}^{1}S}_{0}{-}^{1}{P}_{1}$ transition. Monte Carlo simulations indicate that a 461‑nm photon scattering rate ${\gamma }_{461}^{({{\rm{out}}})}=30\,{{{\rm{s}}}}^{-1}$ reproduces this additional heating-induced loss $\Delta {\gamma }_{{{\rm{S}}}}$. For the Fig. 5 spectrum, no additional post-processing correction was applied to the experimental data because the measured out-of-chamber loss rates, ${\gamma }_{{\rm{S}}}^{({{\rm{out}}})}$ and ${\gamma }_{{\rm{P}}}^{({{\rm{out}}})}$, are nearly equal for the two states (see Fig. 6c).

### State-selective detection

After interrogation, atoms continue to be transported for 750 ms, corresponding to a distance of 12 mm, to the imaging region located above the spectroscopy chamber. The number of atoms ${N}_{\rm{g}}$ in the ${{}^{1}S}_{0}$ ground state is measured by fluorescence on the ${{}^{1}S}_{0}{-}^{1}{P}_{1}$ transition at 461 nm, using near-resonant probe beams. We used Probe 1 with a diameter of ≈0.4 mm, aligned with the lattice axis, for imaging the spatial profile of atoms along the axis (Figs. 2 and 3), and Probe 2, with a beam diameter of ∼1 mm, for spectroscopy (Fig. 5). After applying a blow-away pulse to remove ${{}^{1}S}_{0}$ atoms, atoms ${N}_{\rm{e}}$ in the ${{}^{3}P}_{0}$ state are optically pumped back to ${{}^{1}S}_{0}$ state by applying 679- and 707-nm lasers and subsequently detected via fluorescence at 461 nm. These procedures enable state-selective detection to determine excitation probability ${P}_{\rm{e}}={N}_{\rm{e}}/({N}_{\rm{g}}+{N}_{\rm{e}})$. Fluorescence is collected using an EMCCD camera, and the recorded images are integrated over the transverse direction (${|x|}$ <0.1 mm) to obtain one-dimensional population profiles along the transport axis, which directly reveal the spatial distribution of atomic populations as shown in Fig. 3a.

### Data analysis and dimensionless coupling constant ${{\boldsymbol{\xi }}}$

**Fig. 7:**
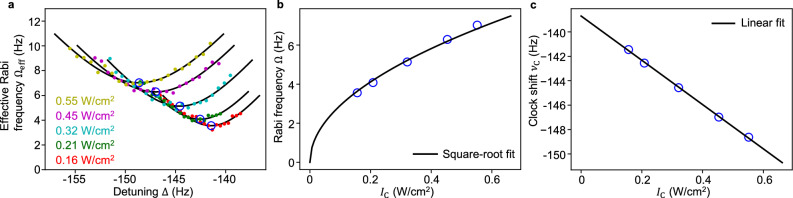
Clock intensity-dependent Rabi dynamics. **a**, Effective Rabi frequencies ${\Omega }_{{{\rm{eff}}}}$ measured for different clock-laser intensities ${I}_{{{\rm{c}}}}$ at a magnetic field of *B* = 2.45 mT. **b**, On-resonance Rabi frequency Ω(B) as a function of clock-laser intensity, exhibiting the expected $\Omega \left(B\right)\propto \sqrt{{I}_{{{\rm{c}}}}}$ scaling for magnetic-field-induced excitation. **c**, Clock shift ${\nu }_{{\rm{c}}}$ as a function of clock-laser intensity. A linear fit yields the clock-light-shift coefficient, which is used to evaluate the light shift under each experimental condition.

To determine the effective Rabi frequency ${\Omega }_{{{\rm{eff}}}}\left(\Delta \right)$, the spatial oscillation of the excited-state signal is fitted with a decaying sinusoid,

1 $${N}_{{\rm{e}}}\left({y}^{{\prime} }\right)=A{e}^{-{a}_{1}{y}^{{\prime} }}\left[1-D{e}^{-{a}_{2}{y}^{{\prime} }}\cos \left(\frac{2\pi {\Omega }_{{{\rm{eff}}}}{y}^{{\prime} }}{u}+\phi \right)\right],$$

with ${y}^{{\prime} }=y-10\,{\mbox{mm}}$. We analyse oscillations in $12\,{\mbox{mm}} < y < 20\,{\mbox{mm}}$ (Fig. 3a), corresponding to the 8-mm-long imaging zone. The extracted ${\Omega }_{{{\rm{eff}}}}\left(\Delta \right)$ exhibits a convex dependence on the detuning, ${\Omega }_{{{\rm{eff}}}}\left(\Delta \right)=\sqrt{{\Omega {\left(B\right)}^{2}+\left(\Delta -{\nu }_{{\rm{c}}}\right)}^{2}}$ as shown by Fig. 3b and Fig. 7a. The on-resonant Rabi frequency scales as $\Omega \left(B\right)=\alpha B\sqrt{{I}_{{\rm{c}}}}$ (see Fig. 3c and Fig. 7b) as expected for magnetic-field-induced excitation. The clock shift ${\nu }_{{\rm{c}}}$ includes the second-order Zeeman shift ${\delta }_{B}=\beta {B}^{2}$ (see Fig. 3d), the clock-light shift ${\delta }_{{\rm{c}}}=\kappa {I}_{{\rm{c}}}$ (Fig. 7c), the BBR shift ${\delta }_{{\rm{BBR}}}$, and stray 461-nm cooling-laser-induced light shift ${\delta }_{461}$.

The clock-laser intensity ${I}_{{{\rm{c}}}}$ was determined from the optical power ${P}_{{{\rm{c}}}}$ measured before the vacuum chamber and the Gaussian beam radius ${r}_{{{\rm{c}}}}=0.60(5)$ mm at the atoms, including the transmission of the intervening optics. The resulting relative uncertainty was 17 %, dominated by the beam-radius measurement. The experimentally obtained clock light shift coefficient is *κ*_exp_ = −17 mHz (mW cm^−2^)^−1^, whose magnitude is one-third smaller than the theoretical value ${\kappa }_{{{\rm{th}}}}$ = −(*α*_P_*−α*_S_)/(2*hϵ*_0_*c*) = −26.5 mHz (mW cm^−2^)^−1^ using polarisabilities ${\alpha }_{\rm{P}}$ and ${\alpha }_{\rm{S}}$ calculated at clock wavelength^49^ (${\epsilon }_{0}$ is the vacuum permittivity and h is the Planck’s constant). This difference is likely due to uncertainty in the effective clock intensity sampled by the atoms. In particular, imperfect overlap between the clock beam and the transported atomic ensemble may have reduced the effective intensity experienced by the atoms below the Gaussian peak intensity inferred from ${P}_{{{\rm{c}}}}$ and ${r}_{{{\rm{c}}}}$. Limited reproducibility of this overlap under the spectroscopy conditions can also contribute.

To reduce sensitivity to this effective-intensity uncertainty, we introduce a dimensionless coupling parameter ${\xi }_{\exp }=\Omega (B)/\sqrt{{\delta }_{B}{\delta }_{{\rm{c}}}}$, which characterises the coupling strength of magnetically induced clock excitation independent of ${I}_{{{\rm{c}}}}$. Across several measurements we obtain ${\xi }_{\exp }=0.21\left(2\right)$. This can be compared with the theoretical value ${\xi }_{{\rm{th}}}=\alpha /\sqrt{\beta {\kappa }_{{{\rm{th}}}}}=0.25$, where α and β are the magnetic-dipole coupling and second-order Zeeman coefficients^29^, and ${\kappa }_{{{\rm{th}}}}$ is the light shift coefficient as discussed above. The experimental value is about 20% smaller than the theoretical value, which is plausibly due to spatial inhomogeneity of the Rabi coupling across the ensemble, arising from clock-intensity gradients and non-uniform magnetic mixing fields within the interaction region; such inhomogeneities reduce the ensemble-averaged Ω(B), and hence the apparent coupling strength.

### BBR shift

We describe the non-isothermal blackbody environment seen by the atoms using four effective surface components, indexed by i={PCB, room, cylinder, wire}, and assign to each an effective solid angle ${\Omega }_{i}$, such that the sum of the four solid angles is 4π.

**Fig. 8:**
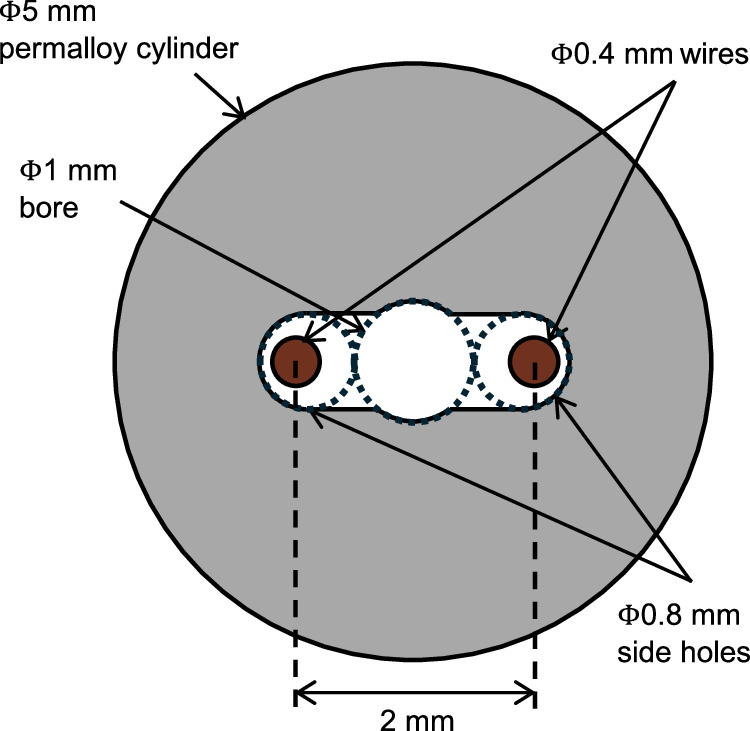
Cross-section of the spectroscopy chamber. The atoms and moving optical lattice pass through the central 1.0-mm-diam. bore. Two 0.8-mm-diam. side holes, centred 0.9 mm from the bore axis, accommodate 0.4-mm-diam. Kapton-coated wires of 2.0 mm apart carrying anti-parallel currents to generate the transverse magnetic mixing field. For the blackbody radiation shift estimate, the central bore is approximated as a cylindrical surface with length L=12 mm and radius r=0.5 mm. Since the side holes are largely occupied by the wires, the cylindrical sidewall contribution is divided into permalloy-cylinder and wire components, with area fractions of 0.87 and 0.13, respectively.

The spectroscopy chamber is approximated as a cylindrical bore of length *L* = 12 mm and diameter 1 mm ($r=0.5\,{{\rm{mm}}}$). Its two apertures face the PCB coil and the room-temperature chamber wall, respectively. The actual structure includes two side holes of diameter 0.8 mm, whose centres are displaced by 0.9 mm from the bore axis, and two Kapton-coated wires of diameter 0.4 mm passing through them (see Fig. 8). Because the side holes are largely occupied by the wires, the cylindrical side-surface area is partitioned between cylinder and wire contributions. The corresponding areas are taken as ${A}_{{{\rm{PCB}}}}={A}_{{{\rm{room}}}}=\pi {r}^{2}$, ${A}_{{{\rm{cylinder}}}}=0.87\times 2\pi {rL}$, and ${A}_{{{\rm{wire}}}}=0.13\times 2\pi {rL}$, with emissivities ${\varepsilon }_{{{\rm{PCB}}}}={\varepsilon }_{{{\rm{room}}}}=1$, ${\varepsilon }_{{{\rm{cylinder}}}}=0.1$, and ${\varepsilon }_{{{\rm{wire}}}}=0.9$.

To estimate the non-isothermal BBR shift, we neglect detailed geometric view factors and take the fractional contribution of each component to be proportional to ${\varepsilon }_{i}{A}_{i}$, with the normalisation chosen so that the four contributions sum to unity. This gives ${\Omega }_{{{\rm{PCB}}}}/4\pi={\Omega }_{{{\rm{room}}}}/4\pi=0.0848$, ${\Omega }_{{{\rm{cylinder}}}}/4\pi=0.354$, and ${\Omega }_{{{\rm{wire}}}}/4\pi=0.476$. The temperatures used in this estimate were obtained in a later run of the same apparatus after thermometry had been added, under operating conditions chosen to reproduce those used for the 1.2-Hz spectrum. ${T}_{{{\rm{PCB}}}}=310(1)\,{{\rm{K}}}$ was measured with a thermistor attached to the tip of the PCB assembly, and ${T}_{{{\rm{room}}}}=298(1)\,{{\rm{K}}}$ with a thermistor attached to the outside of the vacuum chamber near the cavity. ${T}_{{{\rm{cylinder}}}}={T}_{{{\rm{room}}}}+2(2)\,{{\rm{K}}}$ was measured with a PT100 sensor attached to the spectroscopy cylinder. The wire temperature could not be measured directly in vacuum; instead, we estimated ${T}_{{{\rm{wire}}}}={T}_{{{\rm{cylinder}}}}+2(2)\,{{\rm{K}}}$ from the calculated ohmic heating of the current-carrying wires at the operating current used for Fig. 5. The quoted uncertainties were chosen to cover the observed scatter in the sensor readings, as well as imperfect reproduction of the operating conditions between the thermometry and spectroscopy runs. For these parameters, the resulting Sr clock BBR shift is

2 $${\delta }_{{{\rm{BBR}}}}={\nu }_{{{\rm{stat}}}}\mathop{\sum}\limits_{i}\left(\frac{{\Omega }_{i}}{4\pi }\right){\left(\frac{{T}_{i}}{{T}_{0}}\right)}^{4}+{\eta }_{6}\mathop{\sum}\limits_{i}\left(\frac{{\Omega }_{i}}{4\pi }\right){\left(\frac{{T}_{i}}{{T}_{0}}\right)}^{6} \\+{\eta }_{8}\mathop{\sum}\limits_{i}\left(\frac{{\Omega }_{i}}{4\pi }\right){\left(\frac{{T}_{i}}{{T}_{0}}\right)}^{8}+{\eta }_{10}\mathop{\sum}\limits_{i}\left(\frac{{\Omega }_{i}}{4\pi }\right){\left(\frac{{T}_{i}}{{T}_{0}}\right)}^{10},$$

where ${T}_{0}=300\,{{\rm{K}}}$, ${\nu }_{{{\rm{stat}}}}=-2.13023\,{{\rm{Hz}}}$, and the dynamic coefficients are ${\eta }_{6}=-0.12998\,{{\rm{Hz}}},{\eta }_{8}=-0.01211\,{{\rm{Hz}}}$, and *η*_10_ = −0.00844 Hz (ref. ^50^). This yields ${\delta }_{{{\rm{BBR}}}}=-2.33(6)\,{{\rm{Hz}}}$. The evaluated shift is only weakly sensitive to reasonable variations in the component weights, indicating that the simplified treatment is adequate for the present BBR uncertainty evaluation.

### 461-nm cooling-laser-induced light shift and decoherence

**Fig. 9:**
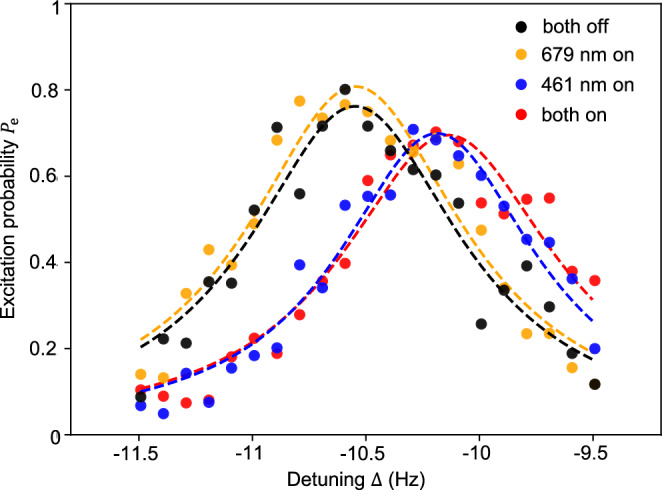
Clock spectra measured with and without 461-nm stray light. The 461-nm light was either present (blue and red circles) or blocked (black and orange circles) during clock interrogation. To avoid bias from differences in out-of-chamber transport loss among the four measurement conditions, the spectra were obtained using Probe 1, with the analysis region of interest (ROI) set to *y* = 22.5−23.0 mm, immediately downstream of the magnetic-shield exit. Comparison of the blue and red circles with the black and orange circles indicates a 461-nm-light-induced frequency shift of *δ*_461_ = 0.4 Hz, which is included in Table 1. In contrast, 679-nm stray light has a negligible effect on the clock transition (black/orange circles). The dashed lines show fits to the corresponding data sets.

Unlike conventional sequential operation, we simultaneously apply the Zeeman slower and MOT beams at 461 nm to prepare cold atoms during spectroscopy. To quantify the effect of 461-nm stray light on the clock spectrum, we measured Rabi spectra with and without 461-nm light during interrogation as displayed in Fig. 9. We observed a frequency shift of ${\delta }_{461}=0.4\,{{\rm{Hz}}}$ in the presence of 461-nm light (Table 1). This light shift implies an excitation rate ${\gamma }_{461}=0.98\,{{{\rm{s}}}}^{-1}$ for the ${{}^{1}S}_{0}$ state, causing an additional dephasing of ${\gamma }_{461}/2$. Further elimination of stray light, such as blackening the vacuum chamber walls^33^ and using low-scattering mirrors, will be implemented in the future experimental setup.

### Calculation of the clock spectrum displayed in Fig. 5

Using experimentally obtained decay rates ${\gamma }_{{{\rm{S}}}}=1.03\left(3\right)\,{{{\rm{s}}}}^{-1}$, ${\gamma }_{{{\rm{P}}}}=1.93\left(4\right)\,{{{\rm{s}}}}^{-1}$, the second-order Zeeman shift ${\delta }_{{{B}}}=-4.57\left(3\right)\,{{\rm{Hz}}}$, the clock light shift ${\delta }_{{{\rm{c}}}}=-3.1\left(4\right)\,{{\rm{Hz}}}$, and the coupling constant ${\xi }_{\exp }=0.21(2)$, we calculated the spectrum using a master equation for ${\rho }_{{ij}}$ with i and j corresponding to atomic states S,P,

3 $${\dot{\rho }}_{{\rm{PP}}}=i\pi \Omega \left(B\right)\left({\rho }_{{\rm{SP}}}-{\rho }_{{\rm{PS}}}\right)-{\gamma }_{{{\rm{P}}}}{\rho }_{{\rm{PP}}},$$

4 $${\dot{\rho }}_{{\rm{SS}}}=-i\pi \Omega \left(B\right)\left({\rho }_{{\rm{SP}}}-{\rho }_{{\rm{PS}}}\right)-{\gamma }_{{{\rm{S}}}}{\rho }_{{\rm{SS}}}+{\gamma }_{{{\rm{R}}}1}{\rho }_{{\rm{PP}}},$$

5 $${\dot{\rho }}_{{\rm{SP}}}=i\pi \Omega \left(B\right)\left({\rho }_{{\rm{PP}}}-{\rho }_{{\rm{SS}}}\right)+i\left[2\pi \left(\Delta -{\nu }_{{{\rm{c}}}}\left(t\right)\right)+i{\gamma }_{\perp }\right]{\rho }_{{\rm{SP}}},$$

where ${\gamma }_{\perp }=({\gamma }_{\rm{S}}+{\gamma }_{\rm{P}}+{\gamma }_{461})/2$ is the transverse relaxation rate, $\Omega \left(B\right)={\xi }_{\exp }\sqrt{{\delta }_{{{B}}}(y){\delta }_{{{\rm{c}}}}}$ is position-dependent Rabi frequency, and ${\nu }_{{{\rm{c}}}}={\delta }_{{{B}}}\left(y\right)+{\delta }_{{{\rm{c}}}}+{\delta }_{{{\rm{BBR}}}}+{\delta }_{461}$ is the total clock shift, with ${\delta }_{{{B}}}(y)$ the position-dependent Zeeman shift obtained from FEM magnetic-field simulation. The atomic motion u converts position dependence $y={ut}+{y}_{0}$ via the time t.

Outside the permalloy magnetic shield ($y > 22\,{{\rm{mm}}}$), the atoms are directly exposed to stray 461-nm light with ${\gamma }_{461}^{({{\rm{out}}})}=30\,{{{\rm{s}}}}^{-1}$ and rapidly lose their coherence with ${\gamma }_{461}^{({{\rm{out}}})}/2$. We also apply ${\gamma }_{{{\rm{S}}}}^{({{\rm{out}}})}=1.7(1)\,{{{\rm{s}}}}^{-1}$ and ${\gamma }_{{{\rm{P}}}}^{({{\rm{out}}})}=1.93(9)\,{{{\rm{s}}}}^{-1}$. Because the loss rates become comparable outside the chamber, the excitation probability measured in Fig. 5 is insensitive to state-dependent loss during transport to the detection region. The resulting spectrum is shown by the dashed line in Fig. 5.

The solid line in Fig. 5 shows the fit to the measurement by taking ${\gamma }_{\perp }=2.3\,{{{\rm{s}}}}^{-1}$ and ${\nu }_{{{\rm{c}}}}=-10.4$ Hz. The mismatch between the linewidths of the experimental spectrum and the calculation may arise from additional dephasing caused by collision shifts, pronounced for bosonic ^88^Sr clocks^25,31^, and from other inhomogeneities in the mixing magnetic field and the clock beam because of the finite temperature of atoms.

## Supplementary information

Transparent Peer Review file

## Data Availability

The EMCCD image data and processed source data underlying Figs. 2, 3, 5–7 and 9 are available in the UTokyo Repository at 10.82337/1000014 (ref. ^51^).

## Code availability

The analysis scripts required to reproduce the plotted data are available in the UTokyo Repository at 10.82337/1000014 (ref. ^51^).
