# Supplementary material for: Spatially defined Rabi spectroscopy for uninterrupted optical clock interrogation
Source: Nat Commun. 2026 Aug 6;17:7662. doi: 10.1038/s41467-026-76017-1 (PMC13448705; doi:10.1038/s41467-026-76017-1)
Supplement: Supplementary file 1 [file 41467_2026_76017_MOESM1_ESM.pdf]

nature portfolio

Peer Review File

Spatially defined Rabi spectroscopy for uninterrupted optical clock interrogation

Corresponding Author: Professor Hidetoshi Katori

This file contains all reviewer reports in order by version, followed by all author rebuttals in order by version.

Version 0:

Reviewer comments:

Reviewer #1

(Remarks to the Author)
The manuscript reports a demonstration of spatially defined optical spectroscopy in a new type of optical lattice clock system. A major upgrade over their previous generations of experiments (Ref. 28, 42) is the addition of electromagnets for clock operation. This enables the spatially-separated, coherent optical excitation of the atoms carried by a continuously moving optical lattice. The authors provide an experimental characterization of systematic effects and dephasing mechanisms in detail. The study of magnetic field dependence is solid and associated clock laser induced Stark shift is also studied. These complete the characterization of magnetic field mixing effect represented by the constant ξ. The data analysis and presentation are largely sound. Overall, this work represents an important step towards the realization of the dead-time free operation an author proposed in Ref. 24, which could potentially be a major impact to frequency metrology and AMO physics in general. We recommend publication in Nature Communications after addressing the following questions and comments.

The state dependent loss rate corrections described in the Methods are important details of this new architecture because it directly changes the deduced excitation fraction as in Fig. 3a. They are mentioned briefly in line 114-117 but we would like to encourage the authors to emphasize their significance and deliver the physical picture of this readout bias mechanism more clearly (state-dependent loss occurring after interrogation but before detection).

The experimental procedure for Figure 4 needs more clarification. On line 169, the authors say "...while scanning the clock-laser detuning...". Is the laser frequency swept continuously or stepped discretely? If it is continuously swept, what is the chirp rate and how should we think about it in terms of the transport speed, u? If the laser frequency is stepped and the signal is acquired at a fixed position/time, the Rabi spectrum in Fig. 4a is not strictly a continuous clock spectroscopy (as in the section title) in a rigorous sense. Also, it is not straightforward to understand how the fixed-time clock spectroscopy time is obtained. The description in line 139 would be more adequately placed under "Continuous clock spectroscopy with Fourier-limited resolution".

We believe having more discussion about the authors' views on the prospect, limitation and challenges of this architecture would be useful for the readers. For example, what would the theoretical Allan deviation of the stabilized laser look like with the current status? Is there a practical or fundamental limit on the Fourier-limited width of 1.2 Hz? For instance, how much light shift would there be due to scattered photons in the detection process? This process will perturb the clock spectroscopy zone, depending on the excitation fraction and the atom number, and the magnitude will scale as 1/d^2. This may set a lower bound on d. There will also be a limitation on u because of the tunneling and/or the physical size of the cavity. This may set an upper bound on u. Finally, is the system truly continuous? We wonder if there is still a small dead time associated with technical details (such as the MOT off period during probing).

Following are some minor comments.

On line 16, the authors mention 'Lamb-Dicke confinement' multiple times and we assume it means η <<1, where η is the ratio between the length scale of the atomic wave packet and the laser wavelength. In our understanding, this is not a necessary condition for clock spectroscopy. As long as the coupling strength between atom and light (both Rabi frequency, 1s Hz, and the decay rates, mHz) is much smaller than any other motional energy gaps along both axial and radial directions (100s kHz to 10s Hz?), precision spectroscopy is possible. In other words, as long as the system is in the resolved sideband regime,

precision spectroscopy is possible. For example, the authors use clock spectroscopy itself to align their clock laser to the lattice axis very precisely and we believe this is possible because the motional energy gap along the radial direction is already larger than the Rabi frequency used. We believe 'Lamb-Dicke' is a term more specific to laser cooling. We do not necessarily ask the authors to change the terminology. However, we would like to hear their view on the use of the term 'Lamb-Dicke confinement' in the context of precision spectroscopy.

How was the atom number calibration for estimating the flux done?

What is the geometry of the triangular cavity? Is the waist located near the MOT?

On line 22, "lattice ensembles" could be 'atomic ensembles' or 'lattice clock ensembles'.

On line 60, could you specify how long the Rayleigh range is?

On line 90, there are two different 461 nm beams introduced: Probe 1 and Probe 2. What is the motivation for having Probe 1? Is it introduced because Probe 2 has a finite beam size?

On line 168, it would be useful to specify which optical pumping beams remained on. Figure 1 contains two optical pumping beams, and clock spectroscopy would not be possible with Optical pump 1 on.

On line 311, "caused by" instead of "responsible for".

The graphic rendering of the figures seems to have issues in MacOS Preview and the print out of it.

Reviewer #2

(Remarks to the Author)
I co-reviewed this manuscript with one of the reviewers who provided the listed reports. This is part of the Nature Communications initiative to facilitate training in peer review and to provide appropriate recognition for Early Career Researchers who co-review manuscripts.

Reviewer #3

(Remarks to the Author)
The authors present the demonstration of continuous spectroscopy on the Sr clock transition in a conveyor-belt lattice - a world-first, to my knowledge. They observe a 1.2 Hz spectroscopic linewidth, in range of the frequency resolution of state-of-the-art optical lattice clocks. In my view, this result will have a major impact in the field of optical clocks, since it opens the door to Dick-effect-free optical lattice clocks with potential frequency instability an order of magnitude below current state-of-the-art. Continuous clocks would also be much less reliant on ultrastable optical cavities, potentially enabling a step change in clock complexity and portability.

The technical content is well explained, the context is well described, and the topics of detailed investigations are well chosen (Rabi flops, decoherence rates & Optical Bloch Eqn model, ac Stark and Zeeman shifts, BBR shifts). I have no hesitation in recommending acceptance for publication in Nature Communications.

I have some comments I would like the authors to address, listed below, but they are all minor. Roughly in order of importance:

1. The authors should explain how the "Excitation probability" data in Figure 4 is generated from raw fluorescence data. At first sight, only the ground state population is actually measured by probe 2. If that is the case, then against what total atom number is the excitation fraction data in Figure 4 normalised?
2. The authors should update the BBR formula with T^8 and T^{10} terms (or acknowledge the existence of these higher-order terms), to avoid reinforcing the common misconception that the dynamic polarizability can accurately be scaled as T^6
3. The authors should explain how the temperatures were measured, and how their uncertainties were evaluated: T_{PCB} = 310(1) K, T_{room} = 298(1) K, $T_{cylinder}$ = T_{room} + 2(2) K, and T_{wire} = $T_{cylinder}$ + 2(1) K
4. Further explanation of the spectroscopy chamber would be helpful, possibly including a drawing of the cylindrical cross-section. The description of the holes into which the wires are fed is a little confusing. At first sight the authors' described geometry would be rather difficult to machine and would result in no solid angle subtended by the wires, contradicting the author's description of the 0.14/0.86 ratio of wire/cylinder contributions to the BBR shift. Also, is the entire spectroscopy chamber made from permalloy, or is there a separate material inserted in the middle guiding the wires?
5. Where the manuscript currently says "The larger loss rate in $^{3}P_{0}$ is responsible for lattice-induced Raman scattering out of $^{3}P_{0}$": Should this say "is due to" instead of "is responsible for"?
6. The author's proposal for continuous excitation-fraction readout is valuable: "Extending the present detection scheme to uninterrupted measurement and feedback should be straightforward. By overlapping the repump beams (679/707 nm) with “Probe 2” (461 nm, beam diameter ϕ=1 mm) and square-wave modulating the repump light at a frequency $f \gg u/\phi$(≈16 Hz), the fluorescence signals can be time-multiplexed to separately obtain the ground- and excited-state populations.". However, could the authors explain why such an approach would be preferable to spatially separated repump after Probe 2, followed by a 461 nm "Probe 3" to measure previously-excited atoms? The latter scheme appears conceptually simpler.
7. If the authors have a full solution in mind to use their continuous spectroscopy scheme to stabilise the frequency of the

laser, it would be very interesting to include such a solution in this manuscript. Their proposed readout scheme is consistent with sitting on one side of the 88Sr clock line to produce a frequency feedback signal, which would crudely allow frequency feedback, but would be vulnerable to systematic shifts and drifts due to lineshape error and instability.
8. The authors should explain how the clock laser intensity I_c = 0.56 W cm^(-2) was determined, and declare its uncertainty. If they have compared the clock probe intensity against expectations from the observed probe ac Stark shift, then it would be interesting to see a comment on this comparison.
9. The colour coding in Figure 3a and 3b would ideally have no overlap, to avoid the possible misinterpretation of there being one-to-one correspondence between the datasets

Version 1:

Reviewer comments:

Reviewer #1

(Remarks to the Author)
The authors have addressed our questions and comments adequately, and we find the revised manuscript significantly improved. The newly added paragraph discussing future prospects is a useful addition that helps readers recognize the remaining challenges and viable routes forward. The new figures are also very helpful, allowing readers to follow the technical details more easily. Furthermore, the newly written descriptions are clear, and we believe they make the manuscript more accessible to readers who are not familiar with this architecture. We look forward to further developments of this architecture and its applications in the field. We have no further comments and recommend the manuscript for publication.

Reviewer #2

(Remarks to the Author)
I co-reviewed this manuscript with one of the reviewers who provided the listed reports. This is part of the Nature Communications initiative to facilitate training in peer review and to provide appropriate recognition for Early Career Researchers who co-review manuscripts.

Reviewer #3

(Remarks to the Author)
I am happy with the authors' responses to my initial review, and with their updates to the manuscript. The authors have made substantial improvements, particularly by adding the figure clarifying the spectroscopy tube dimensions and materials, and by including a clear explanation of the normalised excitation measurement sequence. I am glad to recommend publication with no further changes.

Open Access This Peer Review File is licensed under a Creative Commons Attribution 4.0 International License, which permits use, sharing, adaptation, distribution and reproduction in any medium or format, as long as you give appropriate credit to the original author(s) and the source, provide a link to the Creative Commons license, and indicate if changes were made.
In cases where reviewers are anonymous, credit should be given to 'Anonymous Referee' and the source.
The images or other third party material in this Peer Review File are included in the article's Creative Commons license, unless indicated otherwise in a credit line to the material. If material is not included in the article's Creative Commons license and your intended use is not permitted by statutory regulation or exceeds the permitted use, you will need to obtain permission directly from the copyright holder.
To view a copy of this license, visit https://creativecommons.org/licenses/by/4.0/

Response to the Reviewers

We thank the editor and the reviewers for their careful evaluation of our manuscript and for their constructive comments. We have revised the manuscript extensively to clarify the measurement procedure of Fig. 4, the state-selective readout, the role of state-dependent loss, the blackbody-radiation (BBR) estimate, and the practical prospects and limitations of this architecture. We have also added two new Extended Data figures: a cross-sectional schematic of the spectroscopy chamber (Extended Data Fig. 4) and the timing sequence used to acquire the clock spectrum (Extended Data Fig. 5). In addition, we have revised the Data Availability and Code Availability statements.

Reviewer #1

We thank the reviewer for the positive and thoughtful assessment of our work and for the constructive suggestions. We have revised the manuscript accordingly.

Comment 1

The state dependent loss rate corrections described in the Methods are important details of this new architecture because it directly changes the deduced excitation fraction as in Fig. 3a. They are mentioned briefly in line 114–117 but we would like to encourage the authors to emphasize their significance and deliver the physical picture of this readout bias mechanism more clearly (state-dependent loss occurring after interrogation but before detection).

Response

We agree with the reviewer that downstream state-dependent loss is an important feature of the present architecture and should be explained more clearly. In the revised manuscript, we now state explicitly in the main text that, for the reconstructed spatial profiles of Fig. 3a, state-selective detection is performed downstream of the interaction region, so the measured fluorescence does not directly represent the atom numbers at the end of interrogation. In particular, the larger loss rate of the ^3P0 state during transport would otherwise underestimate the excitation probability, and we therefore correct the reconstructed spatial profiles using independently measured state-dependent loss rates.

At the same time, we now clarify that this issue is different for the Fig. 4 spectrum. Outside the spectroscopy chamber, the measured loss rates are nearly equal, with γ_S^(out) = 1.7(1) s^{-1} and γ_P^(out) = 1.93(9) s^{-1}. This occurs because the additional heating loss induced by stray 461-nm light increases the ground-state loss rate to a level comparable to that of the excited state. As a result, no additional post-processing correction was applied to the Fig. 4 experimental data; the differential relaxation mainly affects the signal amplitude and has negligible effect on the spectral peak position within the present uncertainty. We have clarified this distinction in both the Results and Methods.

Comment 2

The experimental procedure for Figure 4 needs more clarification. On line 169, the authors say "…while scanning the clock-laser detuning…". Is the laser frequency swept continuously or stepped discretely? If it is continuously swept, what is the chirp rate and how should we think about it in terms of the transport speed,

u? If the laser frequency is stepped and the signal is acquired at a fixed position/time, the Rabi spectrum in Fig. 4a is not strictly a continuous clock spectroscopy (as in the section title) in a rigorous sense. Also, it is not straightforward to understand how the fixed-time clock spectroscopy time is obtained. The description in line 139 would be more adequately placed under "Continuous clock spectroscopy with Fourier-limited resolution".

Response

We thank the reviewer for pointing out this ambiguity. The clock-laser frequency was not chirped during acquisition of a single data point. Instead, for each point in Fig. 4, the detuning was set to a fixed value and held constant during acquisition, and the detuning was stepped by 0.1 Hz between measurements. We now state this explicitly in the revised manuscript and have added Extended Data Fig. 5 to show the timing sequence.

We also followed the reviewer's suggestion regarding the placement of the description originally around line 139 of the previous version. In the revised manuscript, the explanation relevant to the downstream Probe-2 readout used for the spectroscopy measurement is now given in the "Clock spectroscopy with Fourier-limited resolution" section, together with the detailed timing sequence shown in Extended Data Fig. 5.

To avoid overstating the nature of the frequency scan, we also changed the section title from "Continuous clock spectroscopy with Fourier-limited resolution" to "Clock spectroscopy with Fourier-limited resolution". The continuous aspect refers to the uninterrupted preparation, transport, and interrogation of the atomic stream at each fixed detuning, rather than to a continuous chirp of the clock-laser frequency.

We also clarify that the 461-nm MOT light is switched off only during the brief Probe-2 fluorescence sequence to suppress stray light on the EMCCD camera. This interruption occurs after clock interrogation and therefore does not remove the 461-nm light shift and decoherence accumulated during excitation.

Comment 3

We believe having more discussion about the authors' views on the prospect, limitation and challenges of this architecture would be useful for the readers. For example, what would the theoretical Allan deviation of the stabilized laser look like with the current status? Is there a practical or fundamental limit on the Fourier-limited width of 1.2 Hz? For instance, how much light shift would there be due to scattered photons in the detection process? This process will perturb the clock spectroscopy zone, depending on the excitation fraction and the atom number, and the magnitude will scale as 1/d^2. This may set a lower bound on d. There will also be a limitation on u because of the tunneling and/or the physical size of the cavity. This may set an upper bound on u. Finally, is the system truly continuous? We wonder if there is still a small dead time associated with technical details (such as the MOT off period during probing).

Response

We agree and have expanded the discussion of the prospects and limitations of the architecture. As already stated in the manuscript, the observed 1.2-Hz linewidth is set by the transit-time Fourier width for the present interaction length $L = 12$ mm and transport velocity $u = 16$ mm s^{-1}. The new discussion added in the revised manuscript concerns the practical constraints relevant to future uninterrupted readout and feedback. In particular, we now discuss the usable feedback bandwidth set by the atomic response time $\tau = L/u$ and the transport delay d/u, as well as the possible perturbation from back-propagating photons generated during future Probe-2 state detection.

We also distinguish two different 461-nm-related effects. The first is the residual Zeeman-slower/MOT stray field present during clock excitation in the current experiment; this is independently characterized by spectra measured with and without the 461-nm light during interrogation and is included in our model. The second is the possible back-propagation of resonant photons generated during Probe-2 state detection in a future fully uninterrupted fluorescence-readout implementation. We now discuss this latter effect as a practical, rather than fundamental, constraint on the detection distance d, and mention possible mitigation strategies including improved baffling, choice of probe polarization, and cavity-enhanced dispersive readout, as cited in the revised manuscript (ref. 34).

Finally, we clarify that the present experiment demonstrates the spectroscopy platform itself. The atomic stream is continuously delivered and interrogated, whereas a fully uninterrupted closed feedback loop has not yet been implemented. The short switch-off of the 461-nm MOT light occurs only during the brief Probe-2 fluorescence sequence and therefore does not define the clock interrogation time.

Comment 4

On line 16, the authors mention 'Lamb-Dicke confinement' multiple times… We would like to hear their view on the use of the term 'Lamb-Dicke confinement' in the context of precision spectroscopy.

Response

We thank the reviewer for this thoughtful comment. We agree that, for precision clock spectroscopy, the more general spectroscopic requirement is that the motional sidebands be sufficiently resolved so that the carrier can be interrogated without appreciable motional broadening.

At the same time, our use of the term "Lamb–Dicke confinement" is not intended as terminology specific only to laser cooling, but rather in the longstanding precision-spectroscopy and frequency-standard sense: confinement tight enough that recoil and first-order Doppler effects are strongly suppressed. This usage is standard in the optical-clock literature. For example, the review by Ludlow et al. states that trapped-ion clocks are typically deep in the Lamb–Dicke regime, thereby eliminating recoil shifts, and that operating in both the resolved-sideband and Lamb–Dicke regimes provides maximum benefit for spectroscopy virtually free of Doppler and recoil effects. The same review also describes optical lattice clocks as benefiting from Lamb–Dicke confinement and traces trapped-ion optical spectroscopy back to Dehmelt's early work. Early neutral-atom lattice-clock papers by Ido and Katori likewise explicitly use the Lamb–Dicke-regime language for recoil-free / Doppler-free spectroscopy in an optical lattice.

In this sense, we believe the terminology is appropriate here and standard in precision spectroscopy, although we agree with the reviewer that the resolved-sideband viewpoint is the more general spectroscopic criterion. We therefore kept the term unchanged. For the reviewer's convenience, the relevant references are: A. D. Ludlow et al., Rev. Mod. Phys. 87, 637 (2015); and T. Ido and H. Katori, Phys. Rev. Lett. 91, 053001 (2003).

Comment 5

How was the atom number calibration for estimating the flux done?

Response

We have clarified this in the Methods. The atom flux was estimated from the fluorescence-calibrated one-dimensional density n_1D(y) multiplied by the transport velocity u. The absolute fluorescence-to-atom-

number conversion was obtained from the known 461-nm scattering rate and the calibrated collection efficiency of the imaging system.

Comment 6

What is the geometry of the triangular cavity? Is the waist located near the MOT?

Response

We thank the reviewer for this point. The lattice-cavity geometry and waist location were already described in the Methods of the original submission. The moving lattice is formed inside a triangular ring cavity with a round-trip length of 200 mm, consisting of two flat mirrors and one concave mirror with a radius of curvature of 150 mm, and the beam waist is located 55 mm from the loading position. To address the reviewer's related request more directly, in the revised manuscript we now also state in the main text that the cavity mode has a Rayleigh range of 71 mm.

Comment 7

On line 22, "lattice ensembles" could be 'atomic ensembles' or 'lattice clock ensembles'.

Response

We have changed this wording in the Abstract to "atomic ensembles".

Comment 8

On line 60, could you specify how long the Rayleigh range is?

Response

We now state in the main text that the lattice-cavity mode has a Rayleigh range of 71 mm. This value was already given in the Methods of the original submission.

Comment 9

On line 90, there are two different 461 nm beams introduced: Probe 1 and Probe 2. What is the motivation for having Probe 1? Is it introduced because Probe 2 has a finite beam size?

Response

We have clarified the different roles of the two 461-nm probe beams. Probe 1 is aligned with the lattice axis and is used to image the spatially encoded Rabi oscillations with longitudinal resolution. Probe 2 is used for downstream spectroscopy readout in Fig. 4. We agree with the reviewer's intuition that Probe 1 is introduced because the transverse Probe-2 geometry is not optimal for directly imaging the longitudinal excitation profile.

Comment 10

On line 168, it would be useful to specify which optical pumping beams remained on. Figure 1 contains two optical pumping beams, and clock spectroscopy would not be possible with Optical pump 1 on.

Response

We agree and have clarified this point in the revised manuscript. The optical pumping beam that remained on during the spectroscopy measurement of Fig. 4 is Optical pump 2. Optical pump 1 is used in the state-selective detection sequence and is not the beam that remains on during clock interrogation.

Comment 11

On line 311, “caused by” instead of “responsible for”.

Response

We have changed the wording accordingly.

Comment 12

The graphic rendering of the figures seems to have issues in MacOS Preview and the print out of it.

Response

We thank the reviewer for pointing this out. We submitted the figures in vector format in a separate file.

Reviewer #2

We thank the reviewer and co-reviewer for their participation in the review process and for their consideration of our manuscript.

Reviewer #3

We thank the reviewer for the very positive assessment of our work and for the helpful suggestions. We have revised the manuscript accordingly.

Comment 1

The authors should explain how the “Excitation probability” data in Figure 4 is generated from raw fluorescence data. At first sight, only the ground-state population is actually measured by Probe 2. If that is the case, then against what total atom number is the excitation fraction data in Figure 4 normalised?

Response

We agree and have revised the manuscript to make this explicit. The $^1S_0$ population is first measured by Probe-2 fluorescence as N_g. After a blow-away pulse removes the remaining ground-state atoms, the $^3P_0$ population N_e is optically pumped back to $^1S_0$ with 679- and 707-nm light and then measured by a second Probe-2 fluorescence pulse. The excitation probability is therefore obtained from state-selective measurements of the two populations, $P_e = N_e/(N_g + N_e)$. We also added Extended Data Fig. 5 to show the detailed timing sequence used in the Fig. 4 measurement.

We now also clarify that no additional post-processing correction was applied to the Fig. 4 experimental data for transport loss between the spectroscopy chamber and Probe 2. Outside the chamber, the measured loss rates are nearly equal, $\gamma_S^{(out)} = 1.7(1)\ s^{-1}$ and $\gamma_P^{(out)} = 1.93(9)\ s^{-1}$, so the differential relaxation has negligible effect on the spectral peak position within the present uncertainty. These measured out-of-chamber loss rates are included in the model calculation shown by the dashed line in Fig. 4.

Comment 2

The authors should update the BBR formula with T^8 and T^10 terms (or acknowledge the existence of these higher-order terms), to avoid reinforcing the common misconception that the dynamic polarizability can accurately be scaled as T^6.

Response

We agree and have revised the BBR section accordingly. In the revised manuscript, we now explicitly include the higher-order dynamic BBR terms proportional to T^8 and T^10 in the expression for \delta_BBR, using the coefficients \eta_6, \eta_8, and \eta_10 from ref. 50. Under our experimental conditions, these higher-order terms modify the total shift only slightly, but we agree that including them explicitly is preferable and avoids implying that the dynamic correction can, in general, be truncated at T^6. The revised evaluation gives \delta_BBR= -2.33(6) Hz.

Comment 3

The authors should explain how the temperatures were measured, and how their uncertainties were evaluated.

Response

We agree and have added this information to the Methods. We now explain that these temperatures were obtained in a later run of the same apparatus after thermometry had been added, under operating conditions chosen to reproduce those used for the 1.2-Hz spectrum. T_PCB and T_room were measured with thermistors attached to the PCB assembly and to the outside of the vacuum chamber near the cavity, respectively. T_cylinder was measured with a PT100 sensor attached to the spectroscopy cylinder. The wire temperature could not be measured directly in vacuum and was estimated from calculated ohmic heating at the operating current. The quoted uncertainties were chosen to cover both the observed sensor scatter and imperfect reproduction of the operating conditions between the thermometry run and the spectroscopy run.

Comment 4

Further explanation of the spectroscopy chamber would be helpful, possibly including a drawing of the cylindrical cross-section.

Response

We have added a new Extended Data Fig. 4 showing the cross-section of the spectroscopy chamber. The figure shows the 1.0-mm central bore for the atoms and lattice, the 0.8-mm side holes, and the 0.4-mm Kapton-coated current-carrying wires. We also revised the BBR Methods section to clarify that the quoted cylinder/wire fractions refer to the simplified partition of the cylindrical side-surface contribution used in the BBR model.

Comment 5

Where the manuscript currently says “The larger loss rate in ^3P0 is responsible for lattice-induced Raman scattering out of ^3P0”: Should this say “is due to” instead of “is responsible for”?

Response

We have changed the wording accordingly.

Comment 6

The author's proposal for continuous excitation-fraction readout is valuable… However, could the authors explain why such an approach would be preferable to spatially separated repump after Probe 2, followed by a 461 nm "Probe 3" to measure previously-excited atoms?

Response

We thank the reviewer for this helpful suggestion. We agree that a spatially separated repump stage followed by an additional 461-nm Probe 3 would be a conceptually simple and viable implementation. In the revised manuscript, however, we chose not to discuss this alternative in detail because our aim is not to identify a unique downstream readout architecture, but rather to demonstrate the spectroscopy platform itself and to outline one straightforward route to uninterrupted state readout based on the existing Probe-2 geometry. To make this intent clearer, the revised text now presents the proposed Probe-2 time-multiplexed readout only as an example rather than as the only possible implementation.

Comment 7

If the authors have a full solution in mind to use their continuous spectroscopy scheme to stabilise the frequency of the laser, it would be very interesting to include such a solution in this manuscript. Their proposed readout scheme is consistent with sitting on one side of the ^{88}Sr clock line to produce a frequency feedback signal, which would crudely allow frequency feedback, but would be vulnerable to systematic shifts and drifts due to lineshape error and instability.

Response

We agree that this is an important point, and we thank the reviewer for identifying it clearly. In the simplest interpretation of our proposed uninterrupted readout, one may operate on a slope of the resonance and use the continuously monitored excitation probability as a feedback signal. However, such a side-of-fringe approach would indeed be susceptible to offsets arising from line-shape distortions and drifts. We therefore decided not to present a definitive locking protocol in the revised manuscript.

Instead, we revised the Discussion to state explicitly that the usable feedback bandwidth is constrained by the atomic response time and by the transport delay between interrogation and detection, and that developing a robust clock-error signal and feedback architecture that avoids offset due to line-shape drifts remains beyond the scope of the present work. We nevertheless expanded the Discussion to explain the main architectural implication: a continuously delivered system can strongly suppress Dick aliasing, and if the dominant local-oscillator noise lies within the usable feedback bandwidth, t^{-1}-like averaging could in principle be approached over an intermediate range of averaging times.

Comment 8

The authors should explain how the clock laser intensity $I_c = 0.56$ W cm^{-2} was determined, and declare its uncertainty. If they have compared the clock probe intensity against expectations from the observed probe ac Stark shift, then it would be interesting to see a comment on this comparison.

Response

We have added a quantitative description of the calibration method to the Methods section. The clock-laser intensity was determined from the measured optical power before the vacuum chamber and the Gaussian beam radius at the atoms, including the transmission of the intervening optics. This gives a relative uncertainty of 17%, dominated by the beam-radius measurement. We also now comment that the experimentally obtained clock-light-shift coefficient is about one-third smaller in magnitude than the theoretical value. Rather than attributing this to the atomic coefficient itself, we interpret it as evidence that the effective clock intensity sampled by the atoms was lower than the Gaussian peak intensity inferred from the independently measured beam size and optical power. A plausible reason is imperfect overlap between the clock beam and the transported atomic ensemble under the spectroscopy conditions, together with limited reproducibility of that overlap. To reduce sensitivity to this effective-intensity uncertainty, we therefore introduce the dimensionless coupling parameter $\xi_{\mathrm{exp}} = \Omega(B)/\sqrt{\delta_B \delta_c}$.

Comment 9

The colour coding in Figure 3a and 3b would ideally have no overlap, to avoid the possible misinterpretation of there being one-to-one correspondence between the datasets.

Response

We agree and have revised Fig. 3 accordingly. We changed the colour scheme used in Fig. 3a so that it no longer overlaps with the palette used in panels b–d, and we now state in the figure caption that the colours in panel a are unrelated to those used in the other panels.
